# Impact of COVID-19 lockdown on flash and real-time glucose sensor users with type 1 diabetes in England

**DOI:** 10.1007/s00592-020-01614-5

**Published:** 2020-10-16

**Authors:** Joshi Prabhu Navis, Lalantha Leelarathna, Womba Mubita, Andrea Urwin, Martin K. Rutter, Jonathan Schofield, Hood Thabit

**Affiliations:** 1 Diabetes, Endocrinology and Metabolism Centre, Manchester University NHS Foundation Trust, Manchester Academic Health Science Centre, Manchester, M13 9WL UK; 2 Division of Diabetes, Endocrinology and Gastroenterology, Faculty of Biology, Medicine and Health, University of Manchester, Manchester,, M13 9WL UK

**Keywords:** COVID-19 lockdown, Flash glucose monitoring, Real-time continuous glucose monitoring, Type 1 diabetes

## Abstract

**Aims:**

People with type 1 diabetes (T1D) face the daily task of implementing self-management strategies to achieve their glycaemic goals. The UK COVID-19 lockdown has had an impact on day-to-day behaviour, which may affect diabetes self-management and outcomes. We assessed whether sensor-based outcomes pre- and during lockdown periods were different in a cohort of glucose sensor users with T1D.

**Methods:**

Data were collected from Freestyle Libre (FSL) or Dexcom G6 sensor users who remotely shared their data with the diabetes clinic web platform. Sensor metrics according to international consensus were analysed and compared between pre-lockdown period and 2 and 3 weeks into lockdown (periods 1 and 2).

**Results:**

Two hundred and sixty-nine T1D patients (baseline HbA1c 57 ± 14 mmol/mol) were identified as FSL (*n* = 190) or Dexcom G6 (*n* = 79) users. In patients with sensor use > 70% (*N* = 223), compared to pre-lockdown period percentage TIR 3.9–10 mM (TIR) significantly increased during period 1 (59.6 ± 18.2 vs. 57.5 ± 17.2%, *p* = 0.002) and period 2 (59.3 ± 18.3 vs. 57.5 ± 17.2%, *p* = 0.035). The proportion of patients achieving TIR ≥ 70% increased from 23.3% pre-lockdown to 27.8% in period 1 and 30.5% in period 2. A higher proportion also achieved the recommended time below and above range, and coefficient of variation in periods 1 and 2. Dexcom G6 users had significantly lower % time below range (< 3.9 mM) compared to FSL users during both lockdown periods (period 1: Dexcom G6 vs. FSL: 1.8% vs. 4%; period 2: 1.4% vs. 4%, *p* < 0.005 for both periods).

**Conclusion:**

Sensor-based glycaemic outcomes in people with T1D in the current cohort improved during COVID-19 lockdown, which may be associated with positive changes in self-management strategies. Further work is required to evaluate long-term sustainability and support.

## Introduction

One of the self-management goals in type 1 diabetes is achieving and maintaining optimal glycaemic control to reduce the risk of complications [1]. This entails the complex daily tasks of frequent monitoring of glucose levels, bolusing insulin at specific times and varying insulin doses according to meal size, physical activities and health status, whilst taking precautionary measures to avoid significant dysglycaemia [2, 3]. The burden of these tasks is compounded by the demands of daily living, causing many people with type 1 diabetes to struggle and experience burnout leading to poor glycaemic outcomes [4–6].

On 23 March 2020, the UK Government implemented movement restriction or lockdown measures in response to the COVID-19 virus global pandemic [7] through the Coronavirus Act, which allowed the UK police to enforce the lockdown [8]. Members of the public, including people with diabetes, were advised to stay at home except for certain “very limited purposes”, such as shopping for basic necessities or for any medical need. Routine outpatient activities were suspended during lockdown as hospitals and healthcare professionals across the UK responded to the pandemic. The lockdown measure has had a unique and widespread impact on societal behaviour, which may have directly or indirectly affected diabetes self-management.

Glucose monitoring technologies, such as flash and continuous glucose systems, are used in routine clinical practice and provide access to real-time glucose values and predictive glucose trends. They also enable remote sharing of glycaemic data between patients and their healthcare professionals through cloud-based web platforms. The benefits of flash and continuous glucose monitoring have been widely published [9–12]. However, their role in evaluating the glycaemic impact of the COVID-19 lockdown measure in the UK is not widely known and may have implications for future diabetes service development.

The objective of this service evaluation was to analyse and compare glycaemic outcomes before and during lockdown in a cohort of people with diabetes using flash and continuous glucose monitors.

## Methods

This was a retrospective, observational, single-centre, service evaluation in a large teaching hospital in the UK. As an audit and service evaluation, no ethical approval was required. Data were collected from outpatient electronic records and glucose monitoring web-based platforms (Libreview; Abbott diabetes care and Dexcom clarity; Dexcom Inc). Patients had provided online informed consent for their data to be remotely connected to and shared with the diabetes clinic.

Sensor data from Libreview and Dexcom clarity were analysed for each of the following periods: pre-lockdown (1–14 February 2020), early lockdown (period 1; 1–14 April 2020) and mid-lockdown (period 2; 1–14 May 2020). Patients were eligible for inclusion if they met the following criteria: history of diabetes, on multiple daily injections (MDI) or insulin pump therapy and using Freestyle Libre flash glucose monitoring (FSL) or Dexcom G6 continuous glucose monitoring on their smartphone device. Analysis was performed in those with at least 70% sensor data available during all 3 periods (pre-, early- and mid-lockdown).

In keeping with international consensus on CGM reporting guidelines [13], we analysed the following glycaemic metrics of each patient using the proprietary reporting function of Libreview and Dexcom Clarity software: % time in glucose range (3.9–10.0 mmol/l) (TIR), % time below range (< 3.9 mmol/l), % time above range (> 10.0 mmol/l), coefficient of variation (CV%) and estimated HbA1c value. Estimated HbA1c (eA1c) values were obtained from each patient’s Libreview and Dexcom clarity account, calculated using a validated equation [14]. Adherence to sensor use was evaluated by assessing % of sensor data availability for both FSL and continuous glucose monitors, and number of daily scans for FSL users.

Data on age, gender, postcode, diabetes duration and type of insulin delivery were collected from outpatient electronic records. Participants were assigned an English index of multiple deprivation rank (IMD, https://www.gov.uk/government/statistics/english-indices-of-deprivation-2019) based upon their postcode. These were then grouped by deprivation decile defined by their position in the ranks from the 32,844 small areas in England subdivided into 10 equal groups, with 1 being the most socio-economically deprived and 10 being the least deprived.

### Statistical analysis

Analyses were performed using paired and unpaired sample *t* tests for normal variable distributions, or Wilcoxon signed rank tests for non-normal variable distributions. The change in TIR for different glucose monitoring systems and insulin delivery modality was adjusted by including baseline TIR as a covariate in the analysis of covariance models. Correlation analysis was performed between laboratory HbA1c and eA1c, TIR, age, duration of diabetes and index of multiple deprivation. Values were presented as mean ± SD or median (interquartile range). The hypothesis testing was ordered at the 0.05 level without any control for multiple testing. We completed analyses with SPSS (IBM software, Hampshire UK, version 25). All *p *values are two-sided.

## Results

Two hundred and sixty-nine type 1 diabetes patients were identified as using FSL (*n* = 190) or Dexcom G6 (*n* = 79) on smartphone devices. Table 1 summarises their baseline characteristics. The majority were using an insulin pump (70%) as their method of insulin delivery. Mean laboratory HbA1c before lockdown announcement was 57 mmol/mol (7.3%).

**Table 1:** Baseline characteristics

Characteristics	Data
Sex, *N* (%)	
Female	123 (46)
Male	146 (54)
Age(years)	41.4 ± 12.9
Duration of diabetes(years)	23.6 ± 12.9
HbA1c mmol/mol	57 ± 14
HbA1c %	7.3 ± 1.3
HbA1c % subgroups	
Multiple daily injections	7.3 ± 1.2
Insulin pump therapy	7.4 ± 1.3
Freestyle Libre	7.3 ± 1.3
Dexcom G6	7.4 ± 1.3
Insulin delivery method, *N*(%)	
Multiple daily injections	80 (30)
Insulin pump therapy	189 (70)
Glucose monitoring system	
Freestyle Libre	190 (71)
Dexcom G6	79 (29)
Index of multiple deprivation rank, median (IQR)	15,369 (8203–25,132)
Index of multiple deprivation decile, median (IQR)	5 (3–8)

Data are mean ± SD, unless specified otherwise. *N* = 269

### *Pre-lockdown sensor analysis *(*n* = 269)

Pre-lockdown mean ± SD TIR was 56.0 ± 18.0%, time above range was 39.1 ± 19.9%, and median (IQR) time below range was 3% IQR 1–6. Twenty-three percent of patients achieved TIR ≥ 70%. Glucose variability pre-lockdown, as measured by sensor glucose coefficient of variation, was 37.3 ± 6.4%. A significant correlation was shown between pre-lockdown laboratory-based HbA1c and sensor-based estimated HbA1c (*r* = 0.67, *p* < 0.005).

There were no differences in % TIR, time above or below range between MDI and insulin pump users. Pre-lockdown % TIR for insulin pump vs. MDI was 56.5 ± 17.5 vs. 55.0 ± 19.1%, *p* = 0.56; time above range was 38.5 ± 19.7 vs. 40.5 ± 20.5%, *p* = 0.46; and median (IQR) time below range was 3.0(1.0–6.0) vs. 3.8(1.0–6.0)%, *p* = 0.47. Median (IQR) sensor use pre-lockdown was high, 97 (91–99) %. No significant difference was observed in the % TIR (55.8 ± 17.2 vs. 56.6 ± 19.9% *p* = 0.74) and time above range (38.6 ± 19.2 vs. 40.4 ± 21.6%, *p* = 0.49) pre-lockdown between FSL and Dexcom G6 users; however, % median (IQR) time below range was significantly lower in Dexcom G6 users (FSL: 4.0 (2–7)% vs. Dexcom: 1.6 (0.5–3.6) %, *p* < 0.005). Among FSL users, the median (IQR) number of scans was 10 (7–14) per day.

### *Sensor data comparison between pre-lockdown and lockdown periods *(*N* = 223)

Two hundred and twenty-three patients (FSL = 152, Dexcom G6 = 71) had at least 70% sensor data available during all 3 periods (pre-lockdown, periods 1 and 2; Table 2). The % TIR (3.9–10.0 mmol/l) during lockdown (periods 1 and 2) was significantly higher compared to pre-lockdown (59.6 ± 18.2 vs. 57.5 ± 17.2%, *p* = 0.002 and 59.3 ± 18.3 vs. 57.5 ± 17.2%, *p* = 0.035, respectively). Hyperglycaemia as measured by time above range was significantly lower in period 1 compared to pre-lockdown (period 1: 35.3 ± 20.2% vs. pre-lockdown: 37.9 ± 18.9%, *p* = 0.001), but was similar to baseline in period 2 (*p* = 0.098). Conversely, glycaemic variability as measured by sensor glucose coefficient of variation was lower during period 2 than pre-lockdown (period 2: 36.0 ± 6.4% vs. pre-lockdown: 37.0 ± 5.8%, *p* = 0.003) but similar during period 1 (*p* = 0.24).

**Table 2:** Sensor-based glycaemic outcomes (paired analysis of the same patient across the 3 time periods)

	Pre-lockdown	Period 1	Period 2	*p*-value (pre-lockdown vs. period 1	*p*-value (pre-lockdown vs. period 2
% Time in range (3.9–10.0 mmol/l)	57.5 ± 17.2	59.6 ± 18.2	59.3 ± 18.3	0.002	0.035
% Time below range (< 3.9 mmol/l)	3.0 (1–6)	4.0 (1–7)	3.0 (1–6)	0.056	0.85
% Time above range (> 10.0 mmol/l	37.9 ± 18.9	35.3 ± 20.2	36.4 ± 20.0	0.001	0.098
Coefficient of variation (%)	37.0 ± 5.8	36.6 ± 6.8	36.0 ± 6.4	0.24	0.003

Data are mean ± SD or median IQR. *N* = 223

No significant change for % time spent below range during both lockdown periods was observed in the whole cohort. Those using the Dexcom G6 CGM, however, had a significantly lower % time below range compared to FSL users during both lockdown periods (Dexcom G6 vs. FSL, period 1 median (IQR): 1.8 (0.4–4.8) vs. 4 IQR (2–9)%. Period 2 median (IQR): 1.4 (0.3–4.4) vs. 4(2–8)%. *p* < 0.005 for both periods).

The proportion of patients achieving clinically recommended ≥ 70% TIR [13] increased from pre-lockdown to periods 1 and 2 (see Table 3). Similarly, the proportion of patients achieving the clinically recommended time below range, above range and coefficient of variation in Table 3 also increased from pre-lockdown to period 2. Thirty-five percent of patients had clinically significant improvement in TIR (change ≥ 5%) during period 2 compared to pre-lockdown, whilst 38% had clinically significant improvement in estimated HbA1c (change ≥ 0.3%). Conversely, there was a deterioration of TIR (change ≥ 5%) in 26% and estimated HbA1c (change ≥ 0.3%) in 23% of patients.

**Table 3:** Proportion of patients achieving recommended sensor-based glycaemic targets

	Pre-lockdown	Period 1	Period 2
% Achieving ≥ 70% time in range	23.3	27.8	30.5
% Achieving ≤ 4% time below range	61.9	57.4	62.3
% Achieving ≤ 25% time above range	26.5	34.5	33.2
% Achieving ≤ 36% coefficient of variation	42.2	43.9	48.9

Data are %. *N* = 223

### Factors associated with improvement and deterioration of TIR.

Table 4 summarises the characteristics of patients whose % TIR improved by ≥ 5% (*n* = 91) or deteriorated by ≥ 5% (*n* = 71) between pre-lockdown and period 2. There was no difference in age, duration of diabetes or index of multiple deprivation rank and deciles between those with improved vs. deteriorated TIR. The improvement in TIR between period 2 and pre-lockdown was negatively associated with baseline pre-lockdown TIR (*r* =  − 0.3, *p* < 0.005) indicating greater improvement in TIR for those with lower TIR at baseline. In keeping with this observation, baseline HbA1c was higher in patients whose % TIR improved by 5% or more.

**Table 4:** Comparison of clinical characteristics of patients with better vs. worse glycaemic control in period 2 compared to pre-lockdown

Characteristics	Time in range change (period 2–pre-lockdown) increased by ≥ 5%	Time in range change (period 2–pre-lockdown) decreased by ≥ 5%	*p*-value
Sex, n (%)			
Female	48 (39)	33 (27)	
Male	51 (35)	38 (26)	
Age	41.0 ± 13.2	41.6 ± 11.9	0.75
Duration of diabetes	23.9 ± 13.3	24.3 ± 13.9	0.85
Time in range, pre-lockdown (%)	48.9 ± 16.9	60.8 ± 15.6	< 0.005
Estimated HbA1c mmol/mol, pre-lockdown	65.3 ± 16.5	57.6 ± 11.4	0.001
Index of multiple deprivation rank, median (IQR)	14,155 (6368– 25,626)	17,687 (9141–25,748)	0.42
Index of multiple deprivation decile, median (IQR)	5 (2–8)	6 (3–8)	0.33

Data are N (%) or mean ± SD unless specified otherwise

There were no differences in % TIR for periods 1 and 2, between FSL and Dexcom G6 or between insulin pump users and MDI. The % TIR for Dexcom G6 vs. Libre in period 1 was 56 ± 20 vs. 61 ± 16%, *p* = 0.06, while for period 2 was 57 ± 21 vs. 60 ± 17%, *p* = 0.14. The % TIR for insulin pump users vs. MDI in period 1 was 60 ± 17 vs. 59 ± 20%, *p* = 0.9, and period 2 was 59 ± 18 vs. 59 ± 18%, *p* = 0.97. Adjusting for baseline % TIR, the change in TIR between pre-lockdown and period 2 was not significantly different for both glucose monitoring systems (FSL and Dexcom G6, *p* = 0.22) and insulin delivery modalities (MDI and insulin pump, *p* = 0.49). No significant correlation was found between absolute TIR in period 1 and period 2, with age, duration of diabetes or index of multiple deprivation rank and deciles.

## Discussion

In this study, we describe the changes that occurred in sensor-based metrics in a cohort of adults with type 1 diabetes who shared their FSL or Dexcom G6 sensor data with our clinical service. We showed that compared to pre-lockdown period, a significant increase in proportion of time spent in range was observed in both lockdown periods accompanied by an increase in the proportion of patients achieving the recommended TIR of ≥ 70%. Over 40% of the cohort had clinically meaningful improvements in both TIR and estimated HbA1c during the lockdown period. Other sensor-based outcomes such as time below range, above range and glycaemic variability were comparable or significantly improved during lockdown period in this cohort.

The global COVID-19 pandemic has placed significant challenges on healthcare systems across the world. Similar to our centre, many healthcare institutions had to suspend usual service provisions such as outpatient clinic appointments to manage the COVID-19 pandemic and had their healthcare workers such as diabetes specialists and educators redeployed to the frontline. A report from Australia showed that reduced access to primary care and hospital services for diabetes, combined with fear of exposure to the virus, had led to a significant drop in access to usual diabetes care [15]. The authors, however, suggested that this risk was mitigated by implementing proactive measures, which included improving access to technologies such as glucose monitoring systems. Use of telemedicine in diabetes care during this pandemic has also been increasing throughout the world including in our own institution, which at present has replaced most face-to-face contacts to minimise exposure and to adhere to social distancing rules. A recent study from India showed that glycaemic control in people with type 1 diabetes worsened during lockdown period mainly due to non-availability of insulin and glucose testing kits [16]. This suggests that different countries with different healthcare systems and resources cope differently in the circumstances.

Other studies from other countries have shown comparable results to ours, suggesting that COVID-19 lockdown measures have not had a deleterious effect on short-term glycaemic control in people with T1D. A study in the north eastern region of Italy involving 33 FSL users with type 1 diabetes analysed sensor-based outcomes during the first 7 days of lockdown [17]. The authors also performed a sub-analysis in a smaller cohort (*N* = 13) who continued working during lockdown. In those who stayed at home, mean and standard deviation sensor glucose, TIR and time above range all improved compared to the weeks before lockdown. However, no changes in glycaemic outcomes were observed in those who continued working. The authors thus speculated that the improvements observed in the former may have likely been due to having more time to focus on diabetes self-management, such as timing of boluses and estimating meal composition, while not being exposed to the demands and stress at the workplace. A Spanish study evaluating 17 Dexcom G5 and 75 FSL users reported significant reductions in TIR, time above range and glucose management indicator (GMI), whilst time below range was unchanged [18]. Dover and colleagues recently analysed paired data from 572 FSL users in Scotland and similarly found improvements in TIR and estimated HbA1c [19]. The authors indicated that socio-economic deprivation was an independent predictor of glycaemic control decline during lockdown.

Our findings advance the observations from the aforementioned Italian and Spanish studies by having a larger sample size, including those on MDI and insulin pump therapy, and comparing 3 time points (pre-lockdown, early and mid-lockdown). Compared to the study from Dover and colleagues, in addition to FSL our analyses also included real-time continuous glucose monitoring users, and no relationship between sensor-based outcomes with the English index of multiple deprivation rank and deciles was found.

Empirical data from our group and others have therefore revealed that in the context of the lockdown period, glycaemic control in people with type 1 diabetes, albeit in the short term, was not significantly compromised and in most cases had improved. A plausible explanation is that the lockdown period provided an opportunity for people with type 1 diabetes to implement appropriate strategies, such as allowing time to bolus before meals and thinking about insulin dose adjustments according to activity levels, without being perturbed or distracted by other daily tasks or unpredictable factors at work. This alteration in behaviour during lockdown may have contributed to the increase in number of patients achieving the recommended sensor-based glycaemic targets [13]. In comparison, national data from the UK Freestyle Libre audit from November 2017 reported a median TIR of only 43% (IQR 27–56%) [20]. Our data show that at least 30% more patients during lockdown were able to achieve clinically meaningful improvements to their TIR and sensor-based estimated HbA1c levels. Whether these are sustainable in the longer term and associated with reduced disease burden is at present unknown. Although an overall TIR improvement was observed, notably 71 patients (32%) had significant TIR deterioration of 5% or more during both lockdown periods. Compared to those with improved TIR, these patients had higher baseline % TIR and lower baseline HbA1c. Due to the retrospective nature of our study, no direct explanation can currently be provided. However, among the plausible reasons for this observation, based on anecdotal patient feedback during clinical consultations, include mental distress associated with loss of income and daily routine, reduced physical activity, increased snacking and weight gain. It is also unclear whether the impact of lockdown has a beneficial or detrimental impact on diabetes management in those without access to sensor-based technology or with type 2 diabetes, although emerging evidence currently suggests the latter [21, 22].

Mean HbA1c at baseline in our cohort was lower compared to the UK national diabetes audit data from England and Wales, which reported mean HbA1c of 64 mmol/mol (8%) for pump users and 71 mmol/mol (8.6%) for those on MDI [23]. This was accompanied by the relatively high median sensor use (> 90%) overall and scanning frequency in FSL users, which suggests that our cohort was motivated. Real-time CGM application in our clinical practice is in keeping with UK NICE recommendations which restrict its application to those with higher hypoglycaemia burden (significant impairment of hypoglycaemia awareness and/or severe hypoglycaemia) [24]. CGM users in our analysis, however, had lower time below range at all 3 time points, highlighting the benefits and value of CGM hypoglycaemia alerts and notifications in this cohort.

To our knowledge, this is the first report from England to show the impact of lockdown measures on glycaemic outcomes in people with diabetes using glucose sensors in clinical practice. Ours is also the largest to date evaluating the impact of lockdown in both real-time (Dexcom G6) and flash glucose monitoring sensor (FSL) users on MDI and insulin pump therapy, thereby widening the scope of our results. Other strengths include the analysis pertaining to professionally recommended glycaemic targets and clinically meaningful improvement in glycaemic outcomes. We acknowledge the limitations of our study. Due to the retrospective nature of the analysis, information related to changes in insulin doses, frequency of contact and advice from healthcare professionals, eating and physical activity behaviours are not available and not collected. Therefore, the factors driving improvements in glycaemic outcomes remain speculative. Our findings were in those using glucose sensors and whose mean baseline HbA1c was lower than the UK national data, therefore limiting generalisability. We also excluded those whose glucose sensors were not linked to their smartphones, to ensure that only consistent data uploads during the lockdown period were available and used in our analysis.

In conclusion, we have shown that sensor-based glycaemic outcomes are improved during lockdown period in people with diabetes equipped with sensor glucose. Further work is required to evaluate its longer-term sustainability, and whether wider access to glucose sensors in the event of future lockdown measures may benefit those without access to this technology at present. This provides further opportunities for future diabetes service development during this global pandemic.
